# Optimizing ^131^I-mIBG Dosimetry: Validation of Simplified Time-Point and Segmentation Approaches

**DOI:** 10.1007/s13139-025-00959-5

**Published:** 2025-11-25

**Authors:** Sara Kurkowska, Małgorzata Poniatowska, Carlos Uribe, Bożena Birkenfeld, Hanna Piwowarska-Bilska

**Affiliations:** 1 Department of Nuclear Medicine, Pomeranian Medical University in Szczecin, Szczecin, Poland; 2 Department of Basic and Translational Research, BC Cancer Research Institute, Vancouver, Canada; 3 Department of Molecular Imaging and Therapy, BC Cancer, Vancouver, Canada

**Keywords:** ^131^I-mIBG, Dosimetry, Segmentation, Single time-point dosimetry

## Abstract

**Purpose:**

To evaluate simplified dosimetry methods for ^131^I-mIBG therapy that maintain agreement with reference method while reducing clinical workflow burdens, particularly beneficial for pediatric applications.

**Methods:**

In 24 patients, we implemented a hybrid protocol combining planar whole-body scans (1, 24, 48 h p.i.) with 24 h SPECT/CT for whole-body and liver dosimetry. Three liver segmentation methods were compared on SPECT/CT: whole-organ manual delineation (reference), 4 mL homogeneous sphere, and 4 mL peak sphere. To estimate time-integrated activity coefficients (TIACs) we modeled biokinetics with monoexponential using: full three-time-point data (reference), reduced combinations (1–2, 1–3, 2–3), Hänscheid single-time-point method, and population-based half-lives. Statistical analysis included linear mixed-effects modeling for segmentation comparisons and Bland-Altman analysis for TIAC method validation.

**Results:**

The median absorbed dose was 0.36 mGy/MBq for liver and 0.09 mGy/MBq for whole-body. Among simplified protocols, dual-time-point 24–48 h imaging demonstrated the least bias, with liver absorbed doses showing − 0.02 mGy/MBq (limits of agreement: -0.08 to 0.04 mGy/MBq) and whole-body absorbed doses 0.001 mGy/MBq (limits of agreement: -0.004 to 0.005 mGy/MBq) compared to the full three-time-point reference results. Among single-time-point methods, for both liver and whole-body, the Hänscheid approach applied at 48 h demonstrated superior performance. The 4 mL peak sphere overestimated absorbed doses by 69% versus whole-organ delineation (0.61 vs. 0.36 mGy/MBq, *p* < 0.001), and homogeneous spheres underestimated by 8% (0.33 vs. 0.36 mGy/MBq, *p* = 0.046).

**Conclusions:**

Clinically feasible ^131^I-mIBG dosimetry can be achieved through: (1) dual 24 h/48 h imaging (< 6% mean bias), (2) single 48 h Hänscheid method (< 6% mean bias), and (3) homogeneous sphere liver segmentation **(**< 8% mean difference from reference).

## Introduction

Iodine-131 metaiodobenzylguanidine (^131^I-mIBG) therapy is a well-established treatment for children diagnosed with neuroblastoma, a high-risk pediatric malignancy. However, by the time patients receive ^131^I-mIBG therapy, they have typically undergone extensive pretreatment, including induction chemotherapy, surgery, and high-dose chemotherapy, which may influence treatment response and toxicity. Personalized dosimetry-guided approaches could theoretically optimize therapeutic outcomes by tailoring radiation absorbed doses to maximize tumor control while minimizing off-target toxicities.

Recent studies have demonstrated the feasibility of dosimetry-guided ^131^I-mIBG therapy. For instance, Cassano et al. and Buckley et al. aimed to deliver a total whole-body absorbed dose of 4 Gy using whole-body dosimetry, maintaining an acceptable safety profile [1, 2]. However, existing data relies primarily on planar imaging (or even Geiger counter placed at least 2 m from the patient), which has been shown to introduce significant variability [3], particularly compared with procedures that incorporate SPECT/CT. Despite enabling three-dimensional activity quantification and improved organ and lesion delineation, SPECT/CT-based dosimetry faces practical challenges in pediatric populations, due to the need for multiple imaging time points, which often require sedation or general anesthesia that add logistical burdens and risks.

To overcome these limitations, there is growing interest in simplified dosimetry approaches that reduce imaging time points while maintaining accuracy. Single timepoint methods such as those proposed by Hänscheid et al. [4] and Madsen et al. [5] have shown promise, though they have been primarily validated for ^177^Lu-based therapies (e.g., ^177^Lu-PSMA and ^177^Lu-DOTATATE) [6, 7] rather than ^131^I-mIBG. Implementing such streamlined protocols could significantly benefit pediatric neuroblastoma patients by minimizing anesthesia exposure and simplifying the clinical workflow.

Another key challenge in dosimetry is segmentation, which remains labor-intensive when performed manually. Artificial intelligence-based segmentation offers a potential solution, however, such methods have not yet been widely implemented in most clinically available software. A simpler alternative is the sphere method [8–10], which requires minimal user input compared to full organ segmentation. This approach has been primarily studied in ^177^Lu therapies for kidneys and tumors and its applicability to ^131^I-mIBG therapy warrants further investigation.

In this study, we examine a cohort of patients who underwent diagnostic ^131^I-mIBG procedures without the need of sedation, to address existing methodological gaps and explore strategies to simplify dosimetry for pediatric patients treated with ^131^I-mIBG. Specifically, we evaluate whole-body, and liver absorbed dose estimates, focusing on reduced time-point imaging protocols and simplified segmentation methods. Whole-body dosimetry is prioritized due to its established association with hematological toxicity [11], a major limiting factor in ^131^I-mIBG therapy. Additionally, the liver is assessed as a key organ at risk, given its significant radiation exposure. By refining and validating these simplified dosimetry approaches, our work aims to contribute to standardized protocols that maintain quantitative reliability while enhancing clinical feasibility, particularly in settings where extensive imaging is impractical.

## Materials and Methods

### Patient Population

This study retrospectively analyzed a subgroup of patients who underwent diagnostic ^131^I-mIBG procedures in Department of Nuclear Medicine of Pomeranian Medical University in Szczecin, Poland. Following injection, each patient underwent a series of 3 whole-body scans and at least one SPECT/CT scan. Activity of ^131^I-mIBG in the syringe was measured pre- and post-injection and noted on the patient chart. Each patient was prescribed thyroid blockade prior to the procedure.

### Image Acquisition

The GE Healthcare NM/CT 850 SPECT/CT system was used for imaging acquisition. For each patient, whole-body planar imaging data was acquired at 1, 24, and 48 h post-injection (p.i.). The system was configured with two detector heads, employing a whole-body scan in the anterior and posterior projections, with a speed of 5 cm/min, matrix size of 1024 × 256 and a pixel size of 2.40 mm. High-energy parallel-hole collimation was employed. Dual energy window scatter correction was applied using an emission window (327.6–400.4 keV) and a lower scatter window (267.3–327.6 keV). No attenuation correction was applied to the planar images. The geometric mean was calculated using the conjugate projections (anterior and posterior views).

SPECT/CT imaging was performed at 24 h p.i. with a total of 60 projections per detector head using a non-circular orbit and a step-and-shoot technique. Each frame had an acquisition duration of 60 s. The system was configured with two detector heads, using a 128 × 128 projection matrix (4.42 × 4.42 mm pixel size). The energy windows used for SPECT acquisitions matched those of the whole-body imaging protocol; CT- standard low dose protocol. Quantitative reconstruction was performed using Hermia software (Hermes Medical Solutions). The reconstruction was performed with ordered subset expectation maximization algorithm using 5 iterations and 10 subsets. The reconstruction included corrections for CT-based attenuation, Monte Carlo-based scatter correction (with 10^9^ simulated photons), and modeling the collimator–detector response and 0.9 cm Gaussian post-filter.

### Segmentation

On the whole-body scans the liver and whole-body were initially segmented on the second time-point images. These segmentations were subsequently transferred to the other time points, repositioned, and reshaped as necessary. For liver evaluation, background correction was carried out by determining the background counts per pixel within the respective background regions of interest (ROIs), which were placed in the upper part of the thigh. The background counts per pixel was then multiplied by the number of pixels in the liver ROI and subtracted from the liver counts to account for tissue located anterior and posterior to the liver. Additionally, we segmented the portion of the body that was within the field of view (FOV) of the SPECT/CT. This allowed us to calculate the ratio of planar counts within the SPECT FOV to the total whole-body planar counts.

Liver segmentation was performed using the CT component of the SPECT/CT to determine the organ mass. The volume of interest (VOI) was then expanded by two SPECT voxels in all directions to account for spill-out activity from the liver. If required, spill-in activity from adjacent organs was manually removed. This method served as the baseline segmentation. Additionally, two 4-mL VOIs were positioned within the liver. The first VOI was placed around the region of the highest activity peak, while the second VOI was positioned in an area with homogeneous uptake, avoiding regions of either the highest or lowest activity. The portion of the body captured within the SPECT/CT FOV was segmented to evaluate the activity within the SPECT FOV. We then applied this SPECT-based activity measurement to scale the corresponding region on the planar image. Finally, using the previously calculated ratio (planar counts within the SPECT FOV to the total whole-body planar counts), we extrapolated from the SPECT region to estimate total whole-body activity.

To assess the reproducibility of homogeneous sphere placement, we additionally performed independent measurements after a 6-month interval, with the second set conducted blinded to the initial results.

### Fitting and Integration Procedures

This study utilized a hybrid dosimetry approach, combining planar imaging to derive time-activity curves with SPECT-based activity quantification for scaling purposes. Initially, time-activity curves (TACs) were fitted using monoexponential function, which were subsequently integrated to determine time-integrated activity coefficients (TIACs). These calculations were performed for both the liver and the whole-body. For curve fitting, we utilized the SciPy Python library [12], employing the Levenberg-Marquardt algorithm for nonlinear least squares fitting. Additionally, for the whole-body reference fitting we added the additional time point at time equal 0 and activity equal to injected activity. TIACs were calculated individually for each patient. Subsequently, using all patient’s data we evaluated the population time-activity curves for the liver and whole-body. To derive population-based time-activity curves, we first computed the percent injected activity per gram (%IA/g) for the liver and whole-body in each patient. Data from all patients were then aggregated to determine the mean and standard deviation of %IA/g at each imaging time point. These population-level data points were subsequently fitted using a monoexponential function, incorporating the standard deviation as the weighting factor, to estimate population-based effective half-lives for the liver and whole-body.

To evaluate the potential for simplified dosimetry methods, we investigated combinations of reduced time points. Specifically, TACs were derived from 1 to 2, 1–3, and 2–3 time-point combinations (instead of the three-time-point approach) and fitted using monoexponential functions. Additionally, the Hänscheid single-time-point method was applied using either the second or third time point. Finally, we used population-based effective half-lives derived in previous step and activity from either the second or third time point.

### Dosimetry

Liver S-value from OLINDA/EXM v2.2.3 (HERMES Medical Solutions) was used to estimate liver self-absorbed dose with the mass adjustment according to patient-specific measurements. In this work, we use the term absorbed dose to refer specifically to the self-absorbed radiation dose. For the 4 mL sphere method, the activity concentration dose factor (ACDF) was derived following the methodology outlined in (10). We multiplied the liver self-dose factors (2.28·10⁻⁵ mGy/(MBq·s) for men and 2.89·10⁻⁵ mGy/(MBq·s) for women) and the organ volumes (1800 g for men and 1400 g for women) an got ACDF of 147.7 mGy·mL/(MBq·h) for men and 145.7 mGy·mL/(MBq·h) for women. For whole-body following [13], we used S-value:

$$S\left(r_{WB}\leftarrow r_{WB}\right)=1.34\cdot10^{-4}m^{-0.921}GyMBq^{-1}h^{-1}$$

where *m* is the patient’s mass in kilograms and the method assumes activity uniformly distributed in the body*.*

### Data Analysis

All analyses were performed using Python (v3.10.12, Python Software Foundation). Quantitative results were summarized as mean ± standard deviation (SD) unless otherwise stated. Simplified time-sampling protocols were evaluated by calculating the percentage differences between TIAC for each method and a reference TIAC for three-time-point method. Results were presented as the percentage deviation to illustrate the variation introduced by each method. Additionally, we used TIAC from each fitting method to estimate absorbed doses and performed Bland-Altman analysis to evaluate agreement between multiple simplified methods and a reference method. For each simplified method, the differences between its absorbed dose estimates and the reference absorbed dose were computed, along with the average of each pair of measurements. The bias was calculated as the mean difference from the reference for each method, and the limits of agreement (LoA) were determined based on the mean difference ± 1.96 times the standard deviation of the differences across all observations. This interval defines the range expected to contain 95% of the differences between methods. We present both aggregated results and individual results for each method. To compare absorbed dose across the three segmentation methods we fitted a linear mixed-effects model with method as a fixed effect and a random intercept for each patient to account for repeated measurements. The model was estimated using Restricted Maximum Likelihood and confirmed to have converged. Normality assumptions were tested using Shapiro-Wilk tests (*p* > 0.05 for all methods). Finally, to compare the reproducibility of the homogenous sphere placement between the two measurements (initial and after 6 months) we performed Bland-Altman analysis to quantify agreement between mean concentration reporting mean bias and 95% LoA. Additionally, we calculated the Pearson correlation coefficient to assess consistency between these two sets of measurements.

## Results

We analyzed 24 patients (mean age: 52.2 years; range: 12.6–73.1 years) who underwent diagnostic ^131^I-mIBG imaging. The cohort consisted of an equal distribution of males (*n* = 12) and females (*n* = 12). The primary clinical indications for imaging included suspected pheochromocytoma (*n* = 16), paraganglioma (*n* = 1), gastroenteropancreatic neuroendocrine tumor (*n* = 1), neuroblastoma (*n* = 1), and malignancy of unknown origin (*n* = 1). Additionally, four patients underwent imaging for follow-up after treatment of neuroblastoma (*n* = 1), pheochromocytoma (*n* = 2), and medullary thyroid cancer (*n* = 1). Among the 24 patients, only two had positive findings, though no hepatic lesions were identified across the cohort. The detected lesions included a neuroblastoma metastasis to the distal femur and a primary pheochromocytoma. SPECT/CT imaging primarily targeted the abdomen, with additional regions included in select cases based on clinical necessity. Notably, three patients exhibited increased thyroid uptake, and follow-up investigations revealed that they had not taken thyroid blockade, despite initially reporting adherence to pre-imaging preparation protocols.

**Fig. 1:**
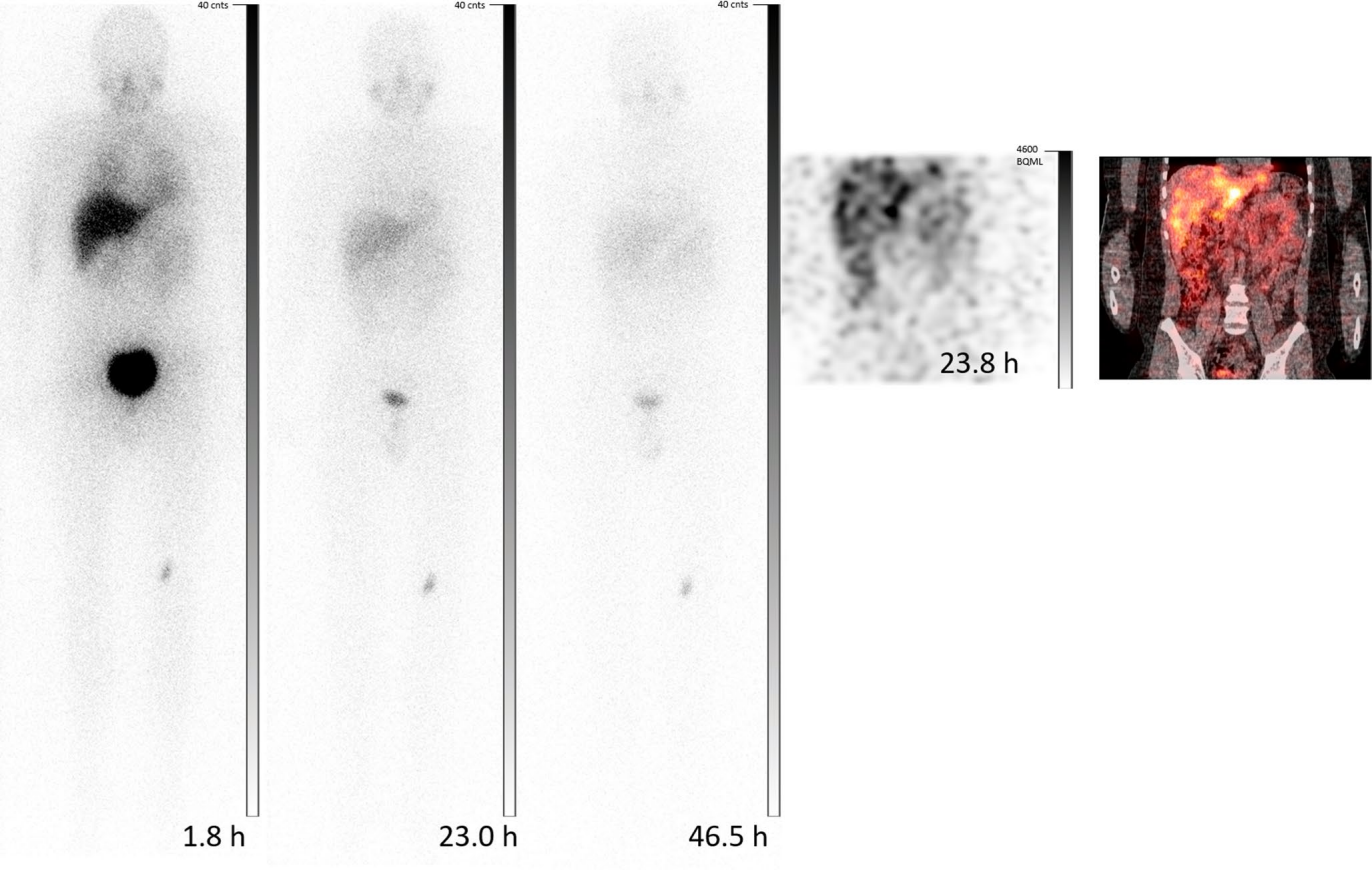
Anterior whole-body planar scans and SPECT/CT images of a 17-year-old patient with previously diagnosed and treated neuroblastoma. The imaging highlights the hybrid approach using planar and SPECT/CT imaging for dosimetry calculations

**Fig. 2:**
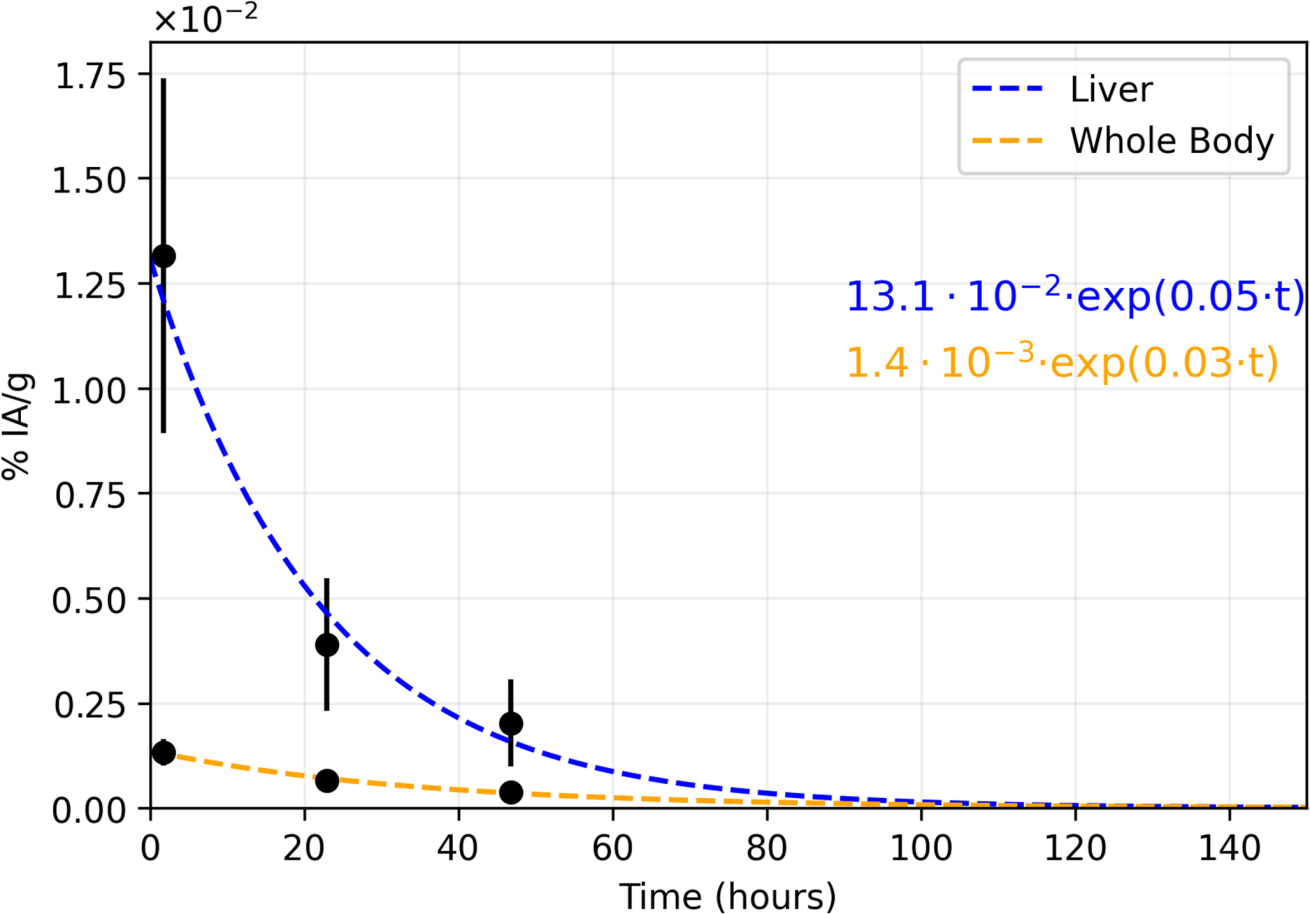
Time-activity curves for the liver and whole-body, expressed as percent injected activity per gram (% IA/g)

**Fig. 3:**
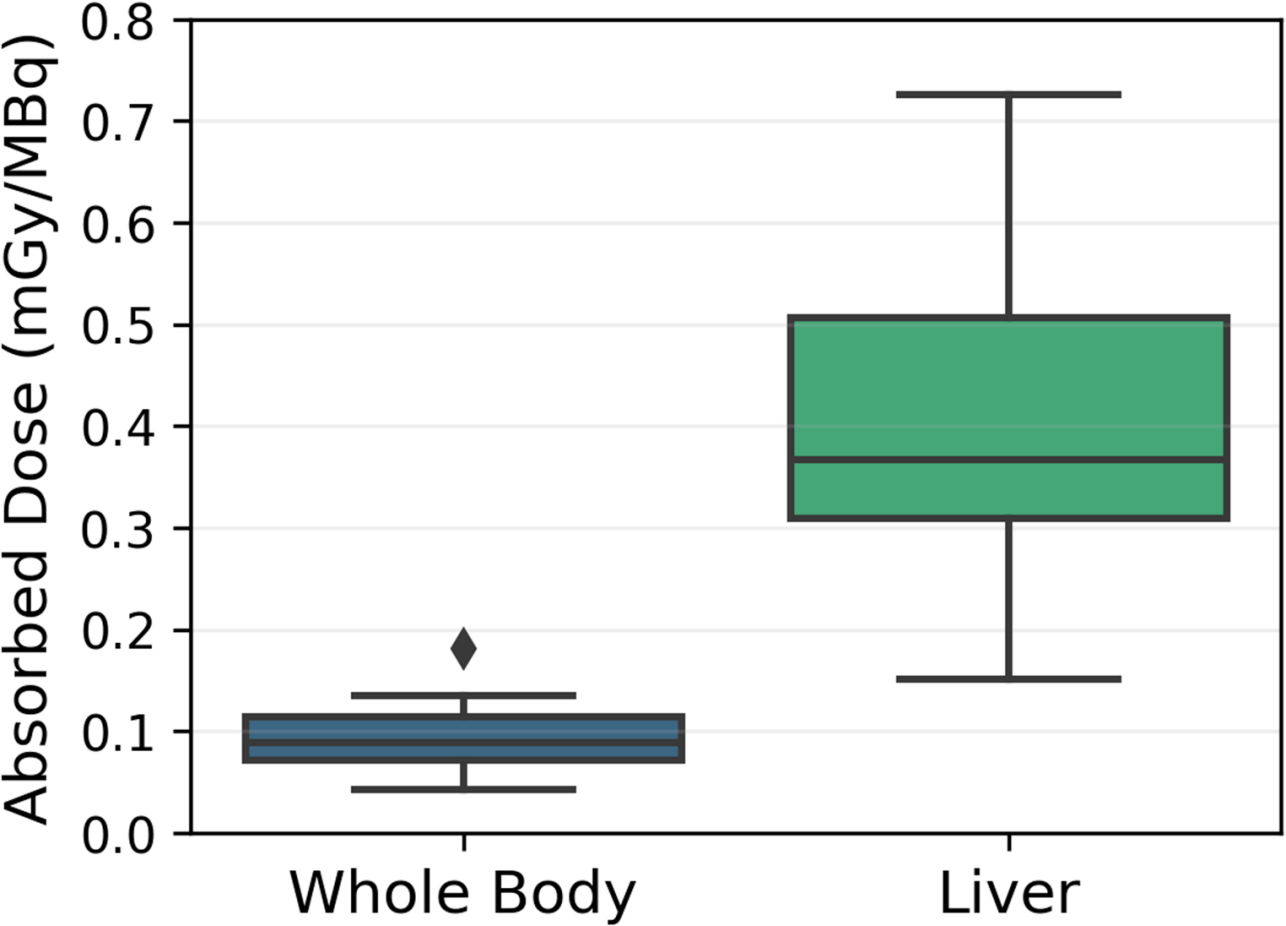
Boxplots showing the distribution of absorbed radiation doses (mGy/MBq) for the liver and whole-body

Figure 1 shows an example patient that undergone planar imaging combined with a SPECT/CT. The population time-activity curves for the liver and whole-body, expressed as percent injected activity per gram (% IA/g) are shown in Fig. 2. Effective half-lives for the liver (15.4 h) and whole-body (24.6 h) were calculated using monoexponential fitting. All the metrics including population liver and whole-body effective decay constants and their corresponding standard errors are presented in Table 1, alongside mean and standard deviation of TIAC and absorbed dose estimates. The boxplots of absorbed doses for the liver and whole-body are shown in Fig. 3.

**Table 1:** Summary of effective decay constant (h⁻¹), time-integrated activity coefficient (TIAC, h), and absorbed dose (mGy/MBq)

	Effective decay constant (h^−1^)	TIAC (h)	Absorbed dose (mGy/MBq)
Liver	0.045 ± 0.011	4.83 ± 1.44 (median: 4.35)	0.41 ± 0.17 (median: 0.36)
Whole body	0.028 ± 0.003	38.69 ± 15.13 (median: 35.26)	0.09 ± 0.03 (median: 0.09)

**Fig. 4:**
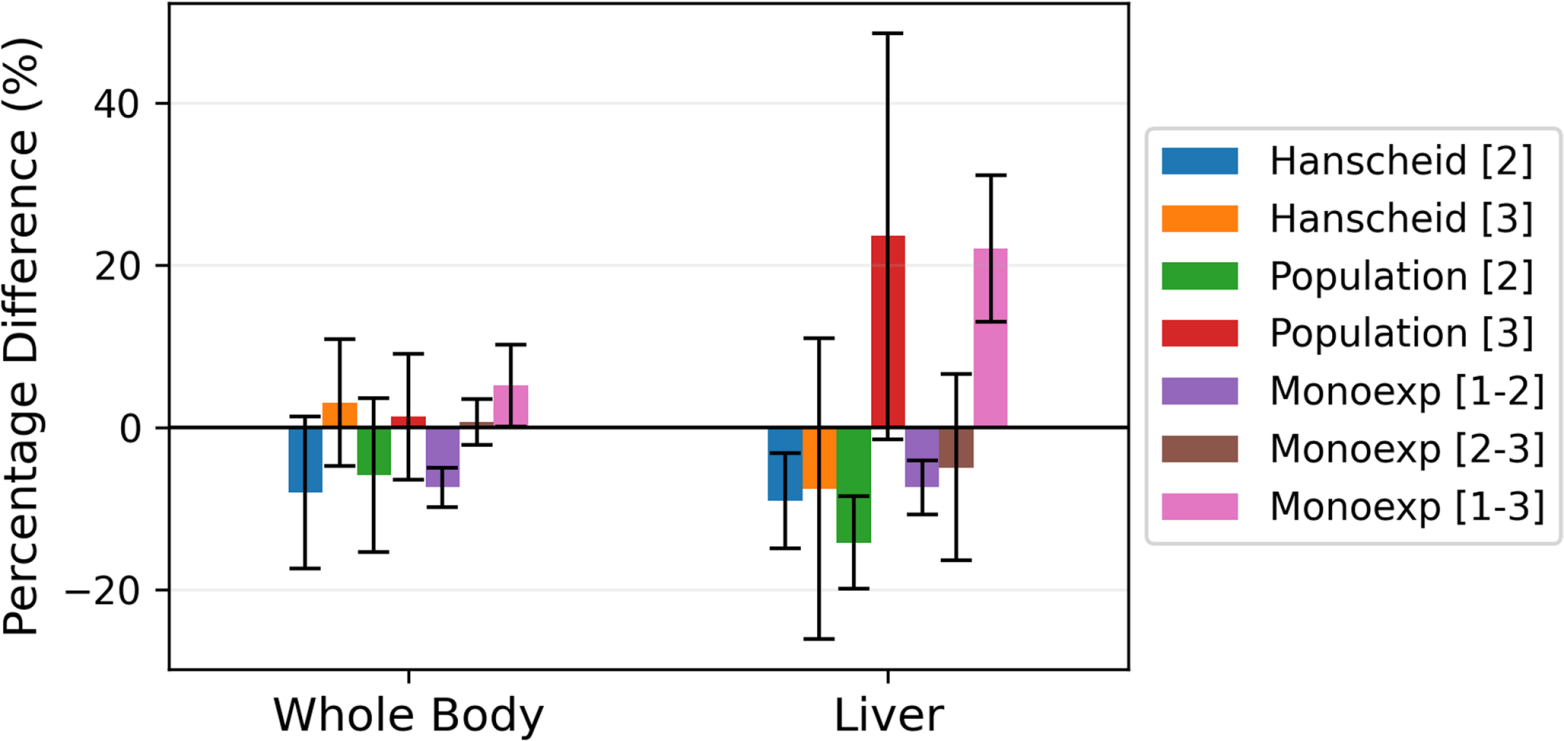
Comparison of percentage differences in time-integrated activity coefficients obtained using reduced time-point fitting methods. These methods include the Hänscheid method and population decay constants applied at time points 2 or 3, and monoexponential fitting using time points 1–2, 2–3 or 1–3. Results are compared to the full three-time-point approach

**Fig. 5:**
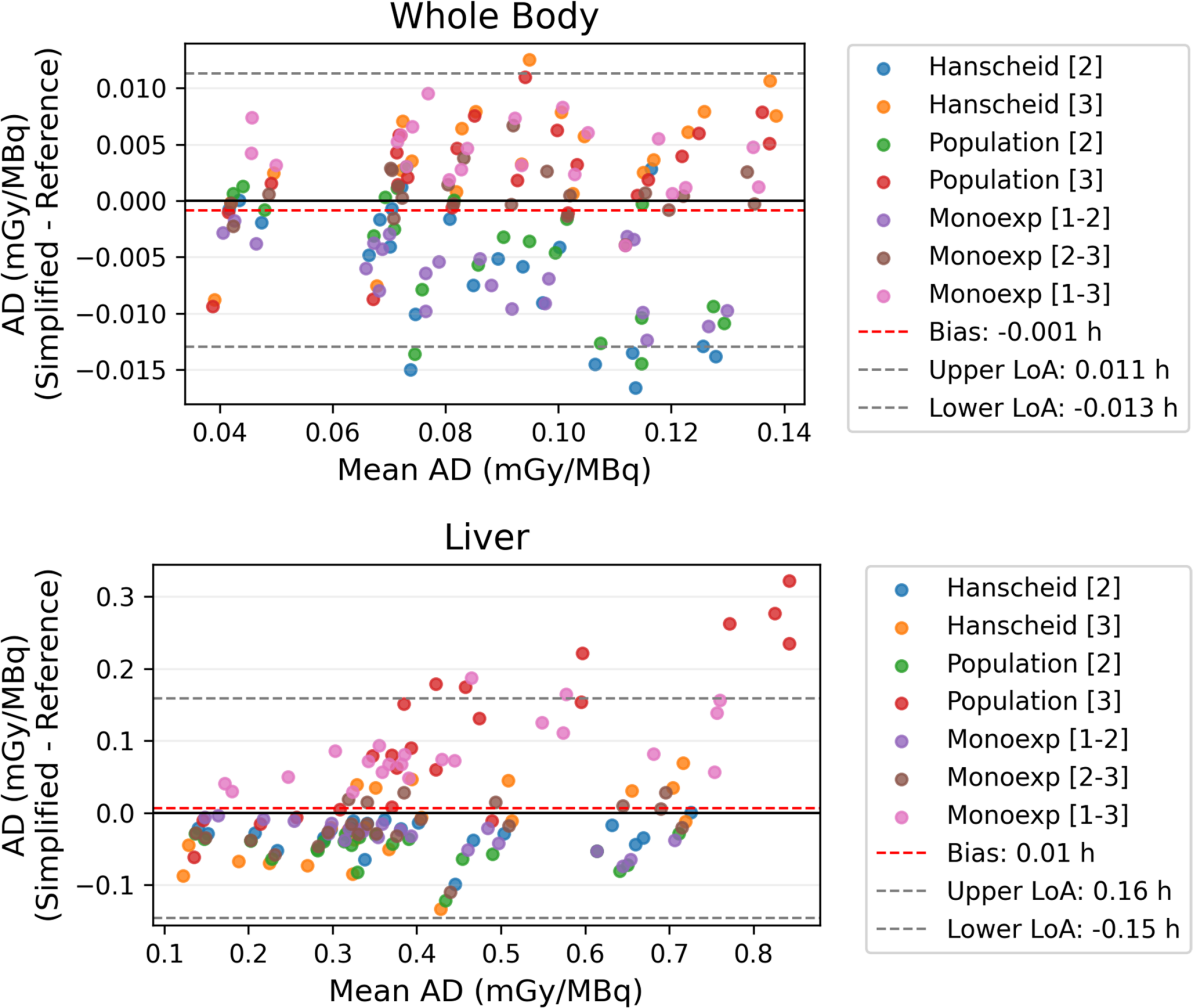
Bland-Altman plots comparing absorbed doses estimated using simplified fitting methods to the reference fitting method for whole-body and liver. Points represent individual differences plotted against the mean of each method pair. Dashed lines indicate general bias and limits of agreement (LoA) for all methods combined. Bias and LoAs for each method are presented in Table 3

When evaluating each patient’s TAC using the reference full three-time-point monoexponential fit (1, 24, 48 h p.i.), the coefficient of determination exceeded 0.98 for the liver and 0.96 for the whole-body across all patients. Figure 4; Table 2 illustrate the percentage differences in TIAC obtained using reduced time-point fitting methods, including the Hänscheid method, population decay constants applied at either time point 2 or 3, and monoexponential fitting using time points 1–2, 2–3 or 1–3, compared to the three-time-point approach (monoexponential 1–2-3 (1, 24, 48 h p.i.)). For the liver, the best agreement with the reference method was achieved with monoexponential method applied to time points 2–3 showing mean − 4.9 ± 11.5% TIAC underestimation, while the most precise estimation came from the monoexponential method applied to time points 1–2 (−7.4 ± 3.3%). In contrast, the poorest performance was associated with the population effective half-life applied at the 3rd time point (23.6 ± 25.0% overestimation). For the whole-body, all methods maintained < 10% deviation from reference values. The monoexponential method applied to time points 2–3 again showed optimal performance, while the Hänscheid method applied at the 2nd time point exhibited the greatest deviation. The results of the Bland-Altman analysis are presented in Fig. 5, with method-specific biases and LoAs detailed in Table 3. Most methods remained within the LoA. However, the population effective half-life applied at the third time point in the liver fell outside the expected limits in the majority of cases suggesting poor performance and indicating high interpatient variability in effective half-life. The monoexponential 2–3 method demonstrated the lowest bias, with absolute differences of 0.001 mGy/MBq (1% of the median whole-body absorbed dose) and − 0.02 mGy/MBq (−5.5% of the median liver absorbed dose) relative to the reference method. For an assumed injected activity of 7400 MBq of ^131^I-MIBG, this would translate to a bias of 7.4 mGy (whole-body) and − 148 mGy (liver). The Hänscheid method at 48 h p.i. yielded biases of 0.004 mGy/MBq (4.4% of the median whole-body absorbed dose) and − 0.02 mGy/MBq (−5.5% of the median liver absorbed dose).

**Table 2:** Comparison of percentage differences (%) in time-integrated activity coefficients obtained using reduced time-point fitting methods

	Hänscheid [2]	Hänscheid [3]	Population [2]	Population [3]	Monoexp [1–2]	Monoexp [2–3]	Monoexp [1–3]
Whole body	−8.0 ± 9.4	3.1 ± 7.8	−5.8 ± 9.5	1.3 ± 7.8	−7.4 ± 2.5	0.6 ± 2.8	5.2 ± 5.0
Liver	−9.0 ± 5.9	−7.5 ± 18.5	−14.2 ± 5.7	23.6 ± 25.0	−7.4 ± 3.3	−4.9 ± 11.5	22.1 ± 9.0

**Table 3:** Bias and limits of agreement (LoA) for each simplified fitting method

	Whole Body			Liver		
Method	**Bias (mGy/MBq)**	**Lower LoA (mGy/MBq)**	**Upper LoA (mGy/MBq)**	**Bias (mGy/MBq)**	**Lower LoA (mGy/MBq)**	**Upper LoA (mGy/MBq)**
Hänscheid [2]	−0.006	−0.018	0.005	−0.03	−0.07	0.01
Hänscheid [3]	0.004	−0.006	0.014	−0.02	−0.12	0.08
Population [2]	−0.004	−0.015	0.006	−0.05	−0.10	−0.01
Population [3]	0.002	−0.007	0.012	0.11	−0.10	0.32
Monoexp [1–2]	−0.006	−0.012	−0.001	−0.03	−0.07	0.01
Monoexp [2–3]	0.001	−0.004	0.005	−0.02	−0.08	0.04
Monoexp [1–3]	0.004	−0.002	0.010	0.09	0.00	0.17

**Fig. 6:**
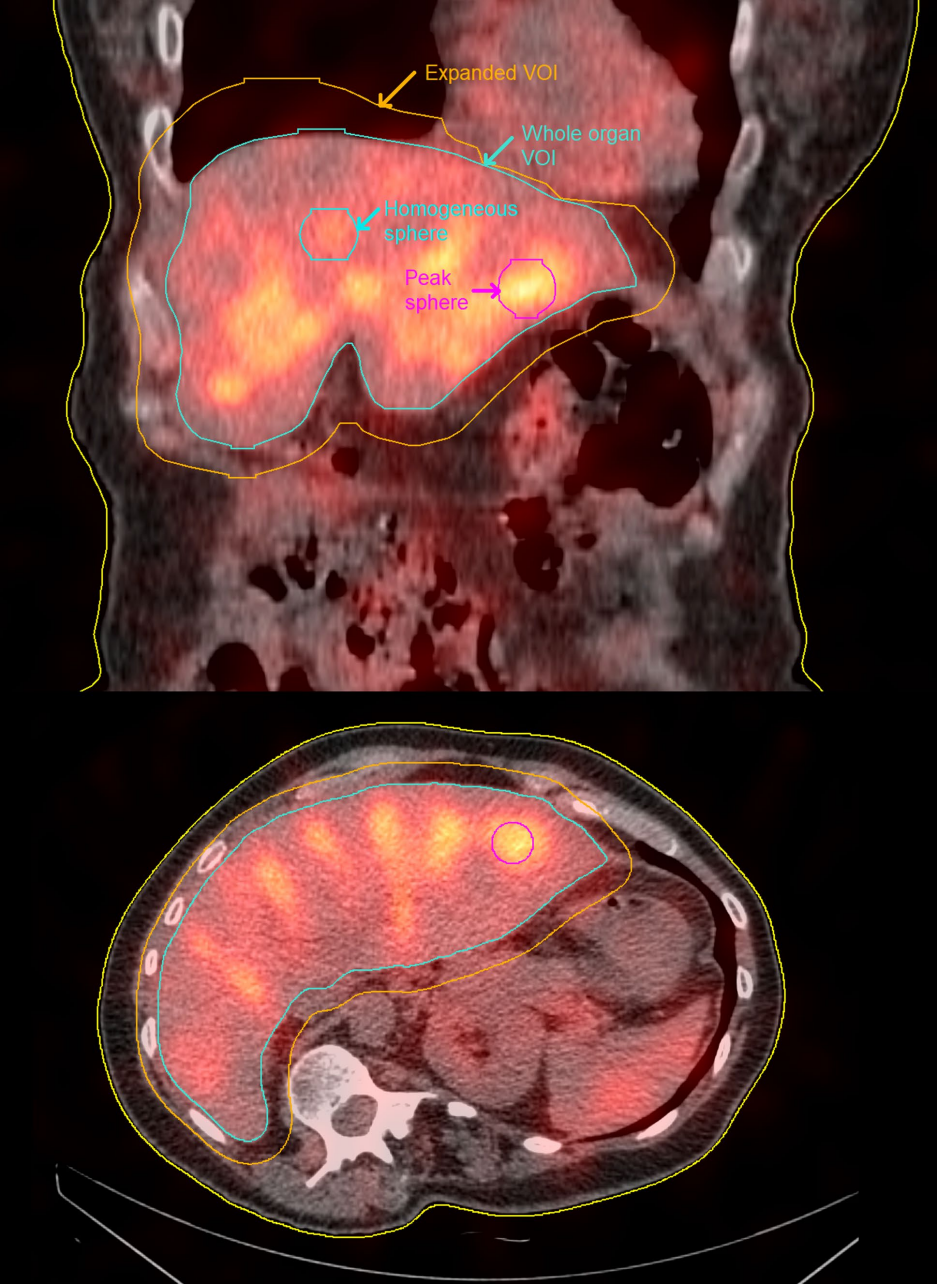
Example of different segmentation methods (whole organ, 4 mL homogeneous sphere, and 4 mL peak sphere)

**Fig. 7:**
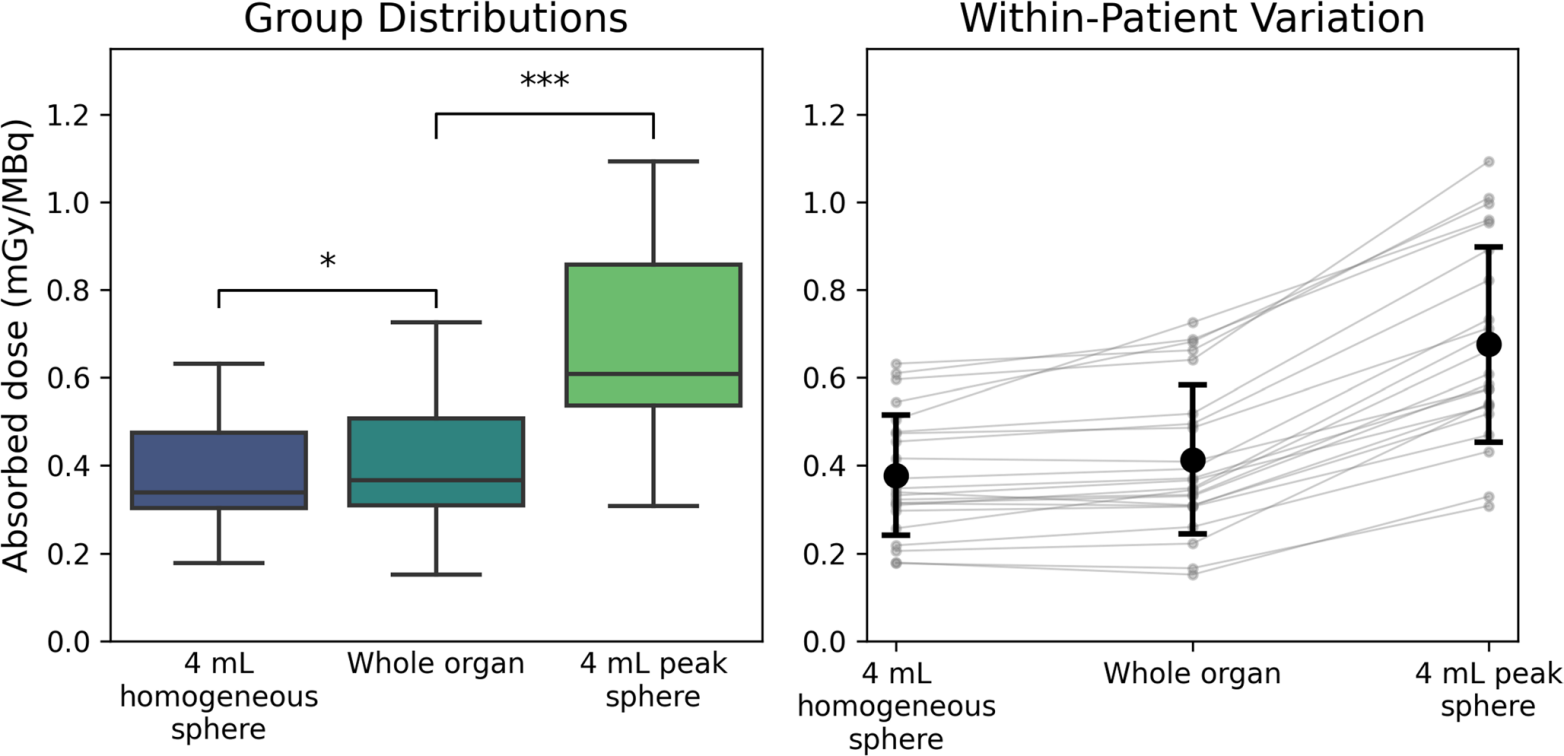
Boxplot illustrating the distribution of absorbed doses for the liver, comparing segmentation methods (whole organ, 4 mL homogeneous sphere, and 4 mL peak sphere) between the group and individual patients

Figure 6 compares different segmentation methods applied to liver VOIs. Quantitative comparison of liver segmentation approaches revealed significant variations in absorbed dose estimates between methods (*p* < 0.001, overall model) (Fig. 7). The median value of the reference whole organ method was 0.36 mGy/MBq. Compared to the whole organ, the 4 mL peak sphere method delivered a 0.26 mGy/MBq higher absorbed dose (95% CI: 0.227–0.297, *p* < 0.001), indicating a substantial increase. In contrast, the 4 mL homogeneous sphere method showed a small but statistically significant reduction of 0.036 mGy/MBq (95% CI: −0.071 to − 0.001, *p* = 0.046) relative to the whole organ. While this difference was statistically significant, its clinical relevance may be negligible given the small effect size.

**Fig. 8:**
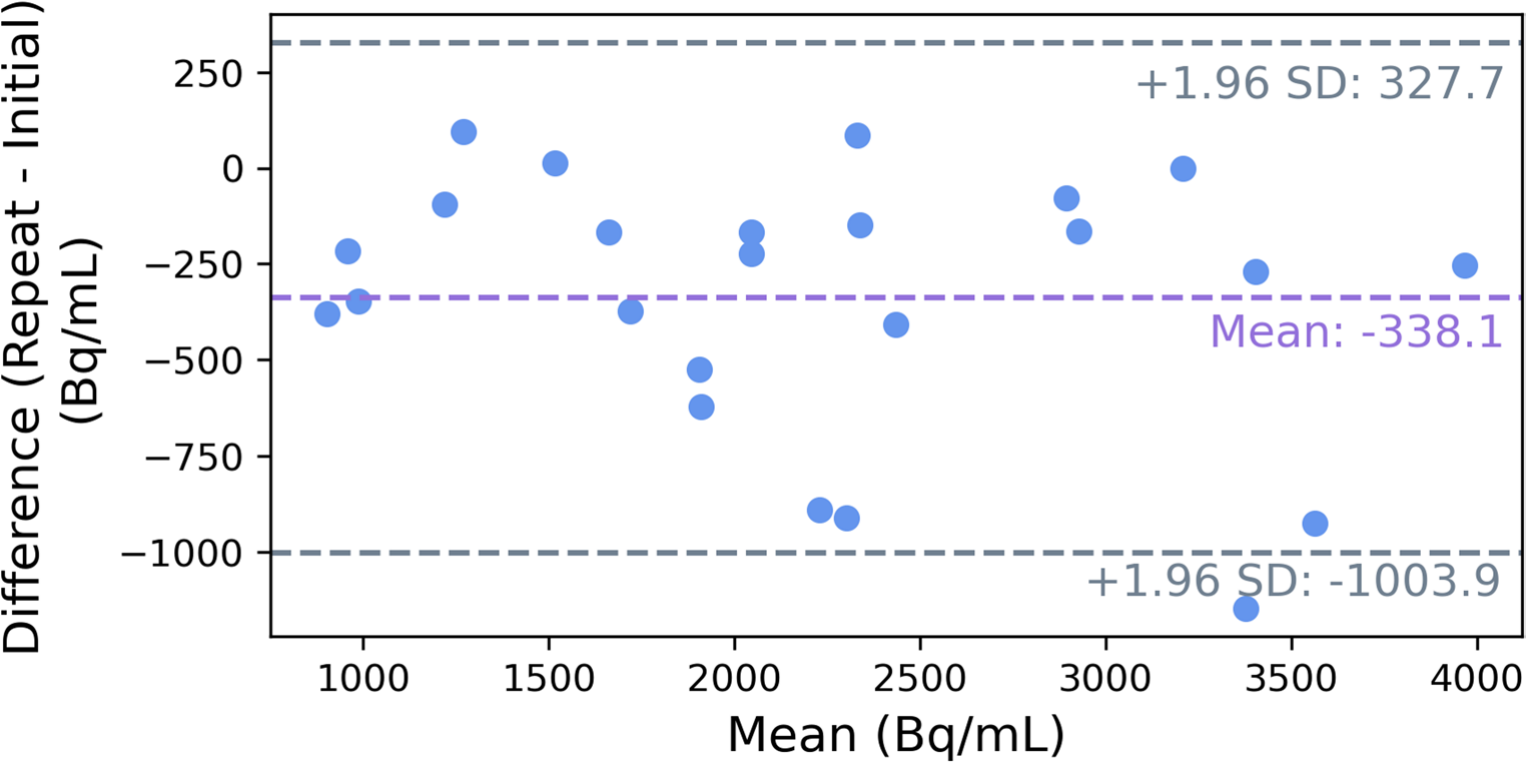
Bland-Altman plots comparing agreement between concentration of radioactivity in initial and repeat placing of homogenous sphere within liver

Analysis of agreement between initial and repeat placing of homogenous sphere is presented in Fig. 8. The mean bias of −338.09 Bq/mL (95% LoA: −1003.9 to 327.7 Bq/mL) corresponds to a −15.3% deviation relative to the average activity concentration across all patients. Repeat measurements were systematically lower with 75% of cases fell within ± 25% of initial values. Pearson correlation coefficient was 0.93 (95% CI: 0.85–0.97), indicating consistency between measurements.

## Discussion

Dosimetry is emerging as a crucial component of personalized treatment in radiopharmaceutical therapies. This study demonstrates that ^131^I-mIBG dosimetry using simplified protocols result in very close estimates to those obtained with multi-time point protocol. This addresses two major clinical challenges: the need for repeated imaging in pediatric patients and labor-intensive segmentation workflows. The validation of reduced time-point approaches and alternative segmentation methods has important implications for implementing personalized therapy in routine clinical practice.

A major barrier to post-therapeutic dosimetry, particularly in neuroblastoma, is the logistical challenge of scheduling and performing multiple imaging sessions. This challenge is amplified in pediatric populations, where imaging often requires sedation or general anesthesia. To address this, we investigated the feasibility of reduced time-point protocols that maintain quantitative reliability while minimizing patient burden. Given these constraints, we adopted a hybrid dosimetry approach. Planar imaging, which can often be performed with caregiver support and without sedation, offers a practical advantage over quantitative SPECT/CT, which requires prolonged stillness and typically necessitates anesthesia in young children. By demonstrating the reliability of simplified imaging protocols, we aim to facilitate broader clinical adoption of dosimetry, even in resource-limited or pediatric settings.

Our absorbed dose estimates, while slightly lower than some literature values, remain consistent with the broader reported ranges. For whole-body absorbed doses, we observed a mean of 0.09 Gy/GBq, which is lower than mean value reported by Sudbrock et al. (0.14 Gy/GBq) [14] and at the lower end of Cassano et al.‘s range (0.04–1.91 Gy/GBq) [1], but shows excellent agreement with Jacobson et al. (0.082 Gy/GBq, range 0.055–0.12) [15]. A similar pattern was observed for liver absorbed doses, where our mean estimate of 0.41 Gy/GBq represents approximately half the values reported by Fielding et al. (0.9 Gy/GBq) [16] and falls below the range reported in [15] (0.50–1.2 Gy/GBq, mean 0.83). These differences may reflect variations in patient physiology and dosimetry methodology, underscoring the importance of institutional-specific absorbed dose evaluations. Additionally, the biodistribution of diagnostic and therapeutic activities can differ. For instance, Hickeson et al. [17] observed that post-therapy scans detected more lesions, and there were differences between uptake in diagnostic and post-therapy scans in normal organs such as nasal mucosa, brain, adrenals, spleen, bowel, thorax and heart.

In our work we evaluated simplified protocols limited to either one or two time points, selecting from the available sampling time points in our dataset (1 h, 24 h, and 48 h p.i.). For protocols limited to two time points, monoexponential fitting using 24 h and 48 h p.i. acquisitions is recommended due to its low bias. Although the estimated biases (7.4 mGy whole-body, −148 mGy liver assuming injected activity of 7400 MBq) appear small clinically, neuroblastoma treatments use widely varying ^131^I-MIBG activities across different protocols, making these differences potentially significant in practice. In single-time-point scenarios, the Hänscheid method proved more reliable than population-based effective half-lives, particularly for liver dosimetry, with optimal performance achieved when using the 48-hour time point (third scan) for both target regions. This observation aligns with the method’s theoretical framework, which maintains accuracy (deviation < 10%) when the ratio of imaging time to effective half-life falls between 0.75 and 2.5 [4]. In our cohort, the measured effective half-lives of 15.4 h for liver and 24.6 h for whole-body placed the 48-hour time point at ratios of 3.1 and 1.95, respectively. While the liver ratio slightly exceeded the upper bound of the recommended range, the results still had < 10% deviation. The consistent superiority of later time points across both dual and single-time-point methods strongly indicates that late-phase imaging better captures the critical elimination phase of ^131^I-mIBG, which is essential for reliable absorbed dose estimation.

The sphere method has been previously validated for kidney and bone marrow dosimetry in several studies. In our investigation, we compared two common sphere placement strategies: positioning around the SUV peak versus placement in regions of homogeneous uptake. Our results demonstrated that absorbed dose from spheres placed in homogeneous activity regions closely matched those derived from whole-organ dosimetry. Although, the difference turned out to be statistically significant, likely due to consistent absorbed dose underestimation for 18 out of 24 patients, it may be negligible in practice. This approach, however, suffers from limited reproducibility due to its subjective, user-dependent nature as shown in our results with the bias of −15.3% for when the same operator performed the sphere placement after 6-months interval. Conversely, the more reproducible SUV peak-based method consistently overestimated absorbed doses by 69% (0.61 vs. 0.36 mGy/MBq, *p* < 0.05). These findings align with previous reports in renal dosimetry. Hou et al., who reported that the sphere method around the highest region overestimated renal absorbed doses by a mean factor of 1.8 [8]. Sandström et al. found that when the sphere method was applied in areas of homogeneous activity (instead of the highest), it underestimated absorbed doses by 8% in the left kidney and 14% in the right kidney compared to whole-organ segmentation [9]. This consistent pattern across studies suggests that while peak-activity spheres introduce systematic overestimation due to sampling high-uptake regions, homogeneous region placement, despite its operator dependence, provides more physiologically representative absorbed dose estimates when properly implemented. Moreover, regardless of the bias we found in comparative analysis between mean concentration measured independently after 6 months, the consistency (Pearson coefficient >0.9) supports the method’s reliability for single-operator workflows.

This study was conducted in a population receiving diagnostic activities of ^131^I-mIBG. Patients were old enough to undergo imaging without sedation and we were able to acquire multiple time points per patient and gain deeper insights into biokinetics without the need for sedation. While these findings provide valuable insights, the extrapolation of results to therapeutic settings, in pediatric neuroblastoma patients, warrants caution. Pediatric dosimetry introduces additional complexities due to differences in radiopharmaceutical distribution, organ sizes, as well as the challenges posed by sedation requirements during imaging. Future studies focusing on pediatric populations receiving therapeutic activities would help validate and extend these findings to a broader clinical context. Additionally, due to the retrospective nature of the study, we had no control over the timing or completeness of voiding between time points. Urine activity accounts for a substantial fraction of the whole-body activity, especially at initial time-points which influences activity estimation and introduces variability in TIAC measurements. The three-time-point method used as the reference was based on imaging at 1, 24, and 48 h p.i. This sampling scheme does not necessarily provide the best description of the radiopharmaceutical biokinetics or the most accurate estimates of TIAC. Ideally, the last time point should be later, at several effective half-lives of the tracer, to capture late washout phase, but this is often very difficult to obtain in clinical practice.

Despite the limitations of this study, our findings suggest that implementing simplified dosimetry approaches for ^131^I-mIBG therapy without compromising quantitative reliability is feasible. The validation of reduced time-point protocols and standardized segmentation methods provides a foundation for more accessible, patient-friendly dosimetry – particularly valuable for pediatric populations where minimizing anesthesia exposure and imaging burden is crucial. As the field moves toward personalized radiopharmaceutical therapy, these practical advancements offer promising steps toward wider clinical adoption of dosimetry-guided treatment optimization.

## Conclusions

Clinically feasible ^131^I-mIBG dosimetry can be achieved through: (1) dual 24 h/48 h imaging (< 6% mean bias), (2) single 48 h Hänscheid method (< 6% mean bias), and (3) homogeneous sphere liver segmentation **(**< 8% mean difference from reference).

## Data Availability

Data availability of data and material: Contact the corresponding author for data requests.

## References

[1] Cassano B, Pizzoferro M, Valeri S, Polito C, Donatiello S, Altini C, et al. Personalized dosimetry for a deeper Understanding of metastatic response to high activity 131I-mIBG therapy in high risk relapsed refractory neuroblastoma. Quant Imaging Med Surg. 2022;12:1299–310. 35111625 10.21037/qims-21-548 PMC8739146

[2] Buckley S, Saran F, Gaze M, Chittenden S, Partridge M, Lancaster D, et al. Dosimetry for fractionated 131 I-mIBG therapies in patients with primary resistant High-Risk neuroblastoma: preliminary results. Cancer Biother Radiopharm. 2007;22:105–12. 17627418 10.1089/cbr.2007.301

[3] Brosch-Lenz J, Ke S, Wang H, Frey E, Dewaraja Y, Sunderland J, et al. Dosimetry challenge an international study of factors affecting variability of dosimetry Calculations, part 2: overall variabilities in absorbed dose. J Nucl Med. 2023;64:1109–16. 37024302 10.2967/jnumed.122.265094 PMC10315703

[4] Hänscheid H, Lapa C, Buck AK, Lassmann M, Werner RA. Dose mapping after endoradiotherapy with ^177^Lu-DOTATATE/DOTATOC by a single measurement after 4 days. J Nucl Med. 2018;59:75–81. 28588150 10.2967/jnumed.117.193706

[5] Madsen MT, Menda Y, O’Dorisio TM, O’Dorisio MS. Technical note: single time point dose estimate for exponential clearance. Med Phys. 2018;45:2318–24. 29577338 10.1002/mp.12886 PMC5948162

[6] Brosch-Lenz J, Delker A, Völter F, et al. Toward Single-Time-Point Image-Based dosimetry of ^177^Lu-PSMA-617 therapy. J Nucl Med. 2023;64:767–74. 36657980 10.2967/jnumed.122.264594 PMC10152120

[7] Hou X, Brosch J, Uribe C, Desy A, Böning G, Beauregard JM, et al. Feasibility of Single-Time-Point dosimetry for radiopharmaceutical therapies. J Nucl Med. 2021;62:1006–11. 33127625 10.2967/jnumed.120.254656 PMC8882881

[8] Hou X, Zhao W, Beauregard JM, Celler A. Personalized kidney dosimetry in 177Lu-octreotate treatment of neuroendocrine tumors: a comparison of kidney dosimetry estimates based on whole-organ and small-volume segmentations. Phys Med Biol. 2019;64:175004. 31456584 10.1088/1361-6560/ab32a1

[9] Sandström M, Garske U, Granberg D, Sundin A, Lundqvist H. Individualized dosimetry in patients undergoing therapy with 177Lu-DOTA-D-Phe1-Tyr3-octreotate. Eur J Nucl Med Mol Imaging. 2010;37:212–25. 19727718 10.1007/s00259-009-1216-8

[10] Del Prete M, Buteau FA, Arsenault F, Saighi N, Bouchard LO, Beaulieu A, Beauregard JM. Personalized ^177^Lu-octreotate peptide receptor radionuclide therapy of neuroendocrine tumours: initial results from the P-PRRT trial. Eur J Nucl Med Mol Imaging. 2019;46:728–42. 30506283 10.1007/s00259-018-4209-7

[11] Buckley SE, Chittenden SJ, Saran FH, Meller ST, Flux GD. Whole-body dosimetry for individualized treatment planning of 131I-MIBG radionuclide therapy for neuroblastoma. J Nucl Med. 2009;50:1518–24. 19713562 10.2967/jnumed.109.064469

[12] Virtanen P, Gommers R, Oliphant TE, Haberland M, Reddy T, Cournapeau D, et al. SciPy 1.0: fundamental algorithms for scientific computing in python. Nat Methods. 2020;17:261–72. 32015543 10.1038/s41592-019-0686-2 PMC7056644

[13] Gear J, Chiesa C, Lassmann M, Gabiña PM, Tran-Gia J, Stokke C, et al. EANM dosimetry committee series on standard operational procedures for internal dosimetry for ^131^I mIBG treatment of neuroendocrine tumours. EJNMMI Phys. 2020;7:15. 32144574 10.1186/s40658-020-0282-7 PMC7060302

[14] Sudbrock F, Schmidt M, Simon T, Eschner W, Berthold F, Schicha H. Dosimetry for ^131^I-MIBG therapies in metastatic neuroblastoma, Phaeochromocytoma and paraganglioma. Eur J Nucl Med Mol Imaging. 2010;37:1279–90. 20179922 10.1007/s00259-010-1391-7

[15] Jacobsson L, Mattsson S, Johansson L, Lindberg S, Fjaelling M. Biokinetics and dosimetry of 131I-metaiodobenzylguanidine (MIBG). Fourth international radiopharmaceutical dosimetry symposium. 1986;389–98.

[16] Fielding SL, FLower MA, Ackery D, Kemshead JT, Lashford LS, Lewis I. Dosimetry of iodine 131 Metaiodobenzylguanidine for treatment of resistant neuroblastoma: results of a UK study. Eur J Nucl Med. 1991;18:308–16. 1936038 10.1007/BF02285457

[17] Hickenson MP, Charron M, Maris J, Brophy P, Kang T, Zhuang H, et al. Biodistribution of Post-Therapeutic versus Diagnostic131I-MIBG scans in children with neuroblastoma. Pediatr Blood Cancer. 2004;42:268–74. 14752865 10.1002/pbc.10454

